# The Prevalence of Bradycardia 12 Years After Roux-en-Y Gastric Bypass for Severe Obesity

**DOI:** 10.1007/s11695-024-07320-3

**Published:** 2024-05-30

**Authors:** Simen Brudeseth, Jorunn Sandvik, Siren Nymo, Gjermund Johnsen, Bård Kulseng, Dag Arne Lihaug Hoff, Torstein Hole

**Affiliations:** 1 Department of Clinical and Molecular Medicine, Norwegian University of Science and Technology (NTNU), 7491 Trondheim, Norway; 2 Obesity Research Group, Department of Clinical and Molecular Medicine, Faculty of Medicine and Health Sciences, Norwegian University of Science and Technology (NTNU), 7491 Trondheim, Norway; 3 Clinic of Surgery, Centre for Obesity, St. Olav’s University Hospital, 7006 Trondheim, Norway; 4 Department of Surgery, Møre Og Romsdal Hospital Trust, 6026 Ålesund, Norway; 5 Clinic of Surgery, Namsos Hospital, Nord Trøndelag Hospital Trust, 7601 Levanger, Norway; 6 National Advisory Unit On Advanced Laparoscopic Surgery, St. Olavs Hospital, Trondheim University Hospital, 7006 Trondheim, Norway; 7 Department of Clinical Studies, Møre Og Romsdal Hospital Trust, 6026 Ålesund, Norway; 8 Department of Health Science Ålesund, Faculty of Medicine and Health Sciences, Norwegian Universiy of Science and Technology (NTNU), 7491 Trondheim, Norway; 9 Medical Department, Ålesund Hospital, Møre Og Romsdal Hospital Trust, 6026 Ålesund, Norway; 10 Faculty of Medicine and Health Sciences, Norwegian University of Science and Technology (NTNU), 7491 Trondheim, Norway

**Keywords:** Bariatric surgery, Gastric bypass, Heart rate, Bradycardia, Weight loss, BMI

## Abstract

**Purpose:**

The aim was to describe the frequency of bradycardia 12 years after Roux-en-Y gastric bypass (RYGB), relations to weight loss, patient characteristics, and the clinical impact.

**Materials and Methods:**

The BAROPS study is a prospective observational study of patients who had follow-up > 10 years after RYGB. Patients with heart rate (HR) ≤ 50 bpm were compared to patients with HR > 50 bpm.

**Results:**

After a mean observation period of 12 years, 32 of 546 patients (6%) had a HR ≤ 50 with a mean HR of 47.0 (2.8) bpm. The comparator group (192 patients) had a mean HR of 66.4 (10.2) bpm (*p* < 0.001). A higher proportion of the bradycardic vs. non-bradycardic patients (18.8% vs. 7.8% at baseline (*p* = 0.05) and 18.8% vs. 5.2% at end of study (*p* = 0.006)) used beta-blockers. Both groups had a significant reduction in heart rate from pre-surgery to end of observation. Percent total weight loss from baseline was negatively related to heart rate (*p* < 0.001), and smoking was positively related to heart rate (*p* = 0.014). Change in BMI from pre-surgery (*p* < 0.001) and hypertension at pre-surgery (*p* = 0.006) were significant predictors of change in heart rate. The only predictor of HR ≤ 50 was the use of beta-blockers (*p* = 0.010). There were no difference in bradycardia-related symptoms.

**Conclusion:**

Six percent of patients had HR ≤ 50 bpm 12 years after RYGB, but there was no increased bradycardia-related symptoms in these patients. RYGB induced a significant reduction in HR, and heart rate and changes in heart rate 12 years after RYGB were related to the amount of weight loss.

**Graphical Abstract:**

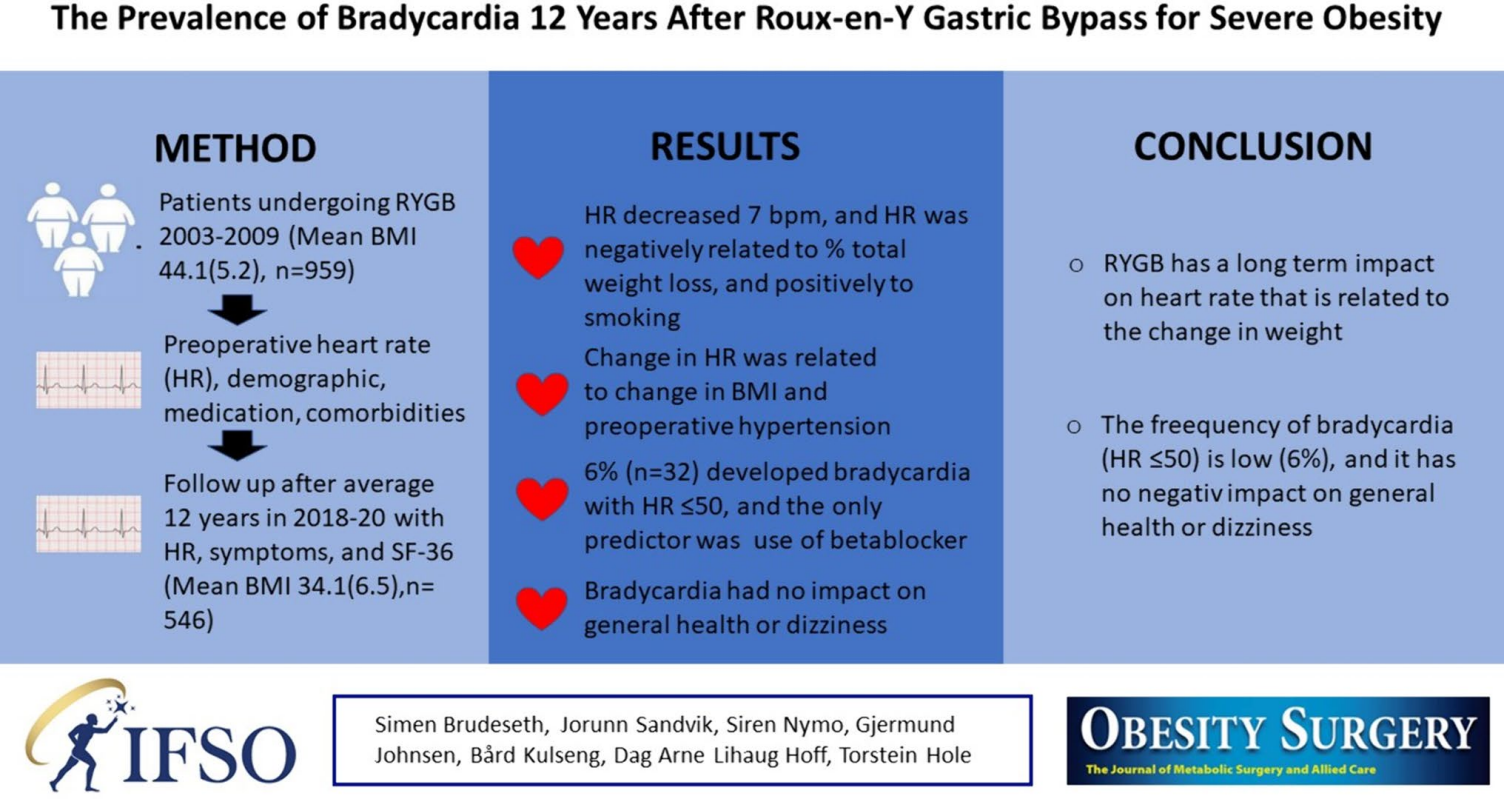

## Introduction

Globally, overweight and obesity are increasing, and there is a well-known relationship between obesity and cardiovascular diseases such as coronary artery disease, heart failure, cardiac arrhythmias, and sudden cardiac death [1–3]. The influence of obesity on diabetes mellitus, hypertension, dyslipidemias, and sleep apnea syndrome leads to an increased incidence of cardiovascular diseases [1–4]. Obesity leads to both functional and structural changes of the cardiovascular system [4, 5]. Several direct and indirect mechanisms are responsible for these changes. Multiple obesity-related mechanisms drive structural, functional, humoral, and hemodynamic changes that increase the risk of cardiovascular diseases [5]. Both obesity and atherosclerosis are considered chronic inflammatory conditions, and there is an activation of both the sympathetic nervous system, the renin–angiotensin–aldosterone system, and hormones such as leptin and adiponectin [5]. Increased heart rate (HR) is only one of many consequences of these changes.

Bariatric surgery reduces the long-term risk of coronary artery disease, cerebrovascular events, and heart failure, and both cardiac structure and function are improved early after bariatric surgery [4, 6]. Significant weight loss is associated with significant changes in hormone levels and alterations in autonomic function [7, 8]. Malik et al. [9] documented the association between bradycardia (HR < 60 bpm) and reduction in BMI due to bariatric surgery. In their study, 25 of 137 (18.2%) patients had postoperative bradycardia at rest with a mean postoperative follow-up period of 17 months [9]. The mean HR postoperatively was 56.7 ± 3.9 bpm (range 46 to 59 bpm) in the bradycardic group. Time to onset or recognition of the first episode of bradycardia was approximately 1–2 years.

The exact physiological mechanism behind bradycardia and changes in HR after bariatric surgery is still not fully understood and needs to be explored. The augmented parasympathetic tone and bradycardia may be due to endogenous neuro-hormonal changes, a decrease in serum leptin, nerve redistribution, or baroreflex impairment [7, 8, 10–14].

Despite that bradycardia generally is asymptomatic and does not require treatment, symptomatic bradycardia may evolve after bariatric surgery [15–17].

The long-term frequency and clinical significance of bradycardia in patients who have undergone Roux-en-Y gastric bypass (RYGB) remain unknown. There are few publications, with more than 5-year follow-up and a high follow-up rate [18]. The lack of comprehensive understanding of this topic, both the change in HR after RYGB and the development of bradycardia, emphasizes the scientific gap and need of more research.

Therefore, the aim of this study was to determine the frequency of bradycardia more than 10 years after RYGB, the clinical impact of bradycardia, and to explore any relations between HR, weight loss, and other selected patient characteristics.

## Material and Methods

### Population

The Bariatric Surgery Observation Study (BAROPS) is a prospective observational study of patients who underwent RYGB at three public hospitals in Mid-Norway between 2003 and 2009 with follow-up in 2018–2020. The aim of the study was to explore long-term changes after bariatric surgery at 10 years of follow-up. Because of some delay in the follow-up program, the mean time was 12 years. Of 959 operated patients, 546 gave informed consent, representing 58.7% of the total. The study was approved by the Regional Committee for Medical and Health Research Ethics of Norway (2017/1828 REK South-East Norway), and all patients gave informed consent. All procedures performed in the study were in accordance with the ethical standards of the national research committee and with the 1964 Helsinki declaration and its later amendments.

The pre-surgery examination is defined as the study baseline, and the BAROPS study examination at the end of the observation period as the end of study (ES).

We defined bradycardia as HR ≤ 50 bpm to ensure that we identified those most likely to have symptoms due to bradycardia. All patients with HR ≤ 50 bpm at ES was included in the study group (SG), and were compared to patients with HR > 50 bpm from one of the three hospitals (RG). There was no significant differences between patients from this hospital and the total population.

**Fig. 1:**
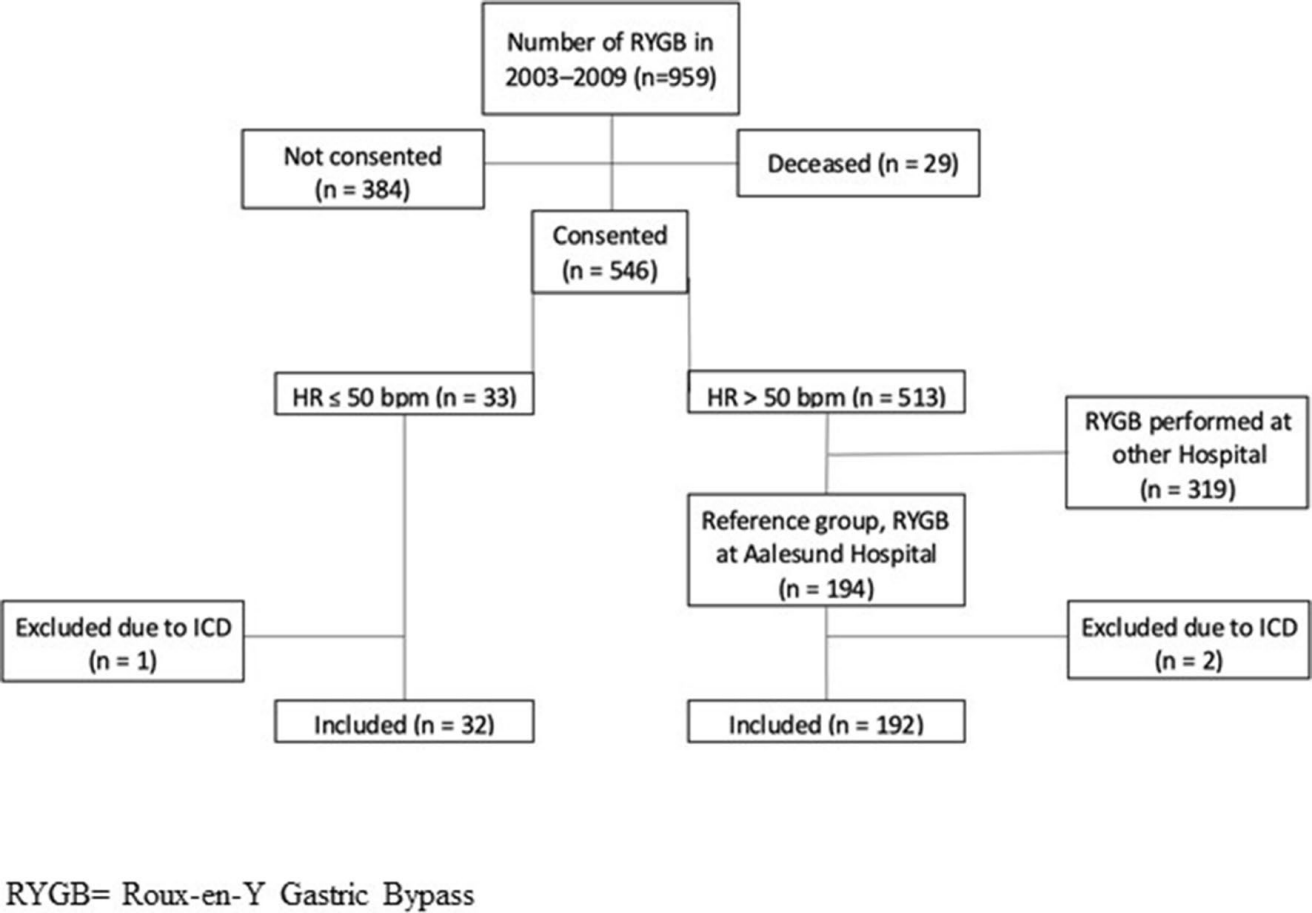
Participant flowchart of the Bariatric Surgery Observation Study

Patients with pacemaker or implantable cardioverter-defibrillator (ICD) implanted between baseline and ES were excluded because the programming of the lower rate behavior of the pacemaker/ICD could decide the allocation to the different study groups and not their intrinsic heart rate. Two patients were excluded in the reference group and one in the study group (Fig. 1).

### Study Parameters

Demographic and anthropometric measurements, blood pressure, HR, ECG, and other parameters were collected at a follow-up control more than 10 years after RYGB (Tables 1 and 2). Information regarding quality of life was retrieved by using 36-Item Short Form Survey (SF-36), a generic instrument consisting of eight domains [19, 20].

**Table 1:** Patient characteristics

	Study group (≤ 50 bpm) (*n* = 32)	Comparator group (> 50 bpm) (*n* = 192)	Total	*p*
Sex ratio (F:M)	20:12 (62.5:37.5)	150:42 (78.1:21.9)	170:54	0.056‡
Age (years)*				
Baseline	40.8 (7.6)	39.6 (9.0)	39.7 (8.8)	0.486†
ES	52.3 (7.5)	51.3 (9.3)	51.4 (9.0)	0.579†
Observation period (months)*	138 (21.2)	140.6 (20.6)	140.2 (20.6)	0.510†
Weight (kg)*				
Baseline	128.2 (17.0)	127.3 (20.4)	127.5 (19.9)	0.828†
Nadir	84.8 (14.5)	82.3 (15.6)	82.7 (15.4)	0.403†
ES	97.4 (22.2)	98.4 (21.4)	98.3 (21.5)	0.817†
BMI (kg/m^2^)*				
Baseline	43.6 (5.4)	44.2 (5.2)	44.1 (5.2)	0.529†
Nadir	28.5 (3.7)	28.5 (4.2)	28.5 (4.1)	0.990†
ES	32.9 (5.7)	34.3 (6.6)	34.1 (6.5)	0.283†
BMI difference (kg/m^2^)*				
Baseline to nadir	15 (4.3)	15.7 (4.3)	15.6 (4.3)	0.440†
Baseline to ES	10.6 (5.9)	9.9 (5.2)	10.0 (5.3)	0.491†
%TWL*				
Baseline to nadir	33.8 (7.2)	35.2 (7.8)	35.0 (7.7)	0.334†
Baseline to ES	24.2 (12.0)	22.8 (11.1)	23.0 (11.3)	0.511†
Hypertension (medicated)				
Baseline	7 (21.9)	42 (21.9)	49 (21.9)	1.0‡
Hypertension (self-reported)	(*n* = 30)	(*n* = 188)	(*n* = 218)	
ES	2 (6.7)	29 (15.4)	31 (14.2)	0.202‡
BP (systolic)*				
Baseline (1 measure)	131.7 (14.5)	130 (13.2)	130.3 (13.4)	0.515†
ES (3 measures)	131.3 (21.9)	128.8 (16.7)	129.2 (17.5)	0.545†
BP (diastolic)*				
Baseline	83.9 (12.0)	82.3 (10.0)	82.5 (10.4)	0.423†
ES	81.8 (11.9)	81.7 (7.6)	81.7 (8.3)	0.951†
Heart rate*				
Baseline	67.6 (14.9)	71.2 (11.2)	70.7 (11.8)	0.194†
ES	47.0 (2.8)	66.4 (10.2)	63.6 (11.6)	< 0.001†
ECG rhythm at baseline	(*n* = 27)	(*n* = 188)	(*M* = 9)	0.704‡
Sinus rhythm	27 (100)	187 (99.5)	214 (99.5)	
Atrial rhythm	0 (0.0)	1 (0.5)	1 (0.5)	
Other rhythm	0 (0.0)	0 (0.0)	0 (0.0)	
ECG rhythm at ES				0.343‡
Sinus rhythm	30 (93.75)	186 (96.9)	216 (96.4)	
Atrial rhythm	2 (6.25)	4 (2.1)	6 (2.7)	
Other rhythm	0 (0.0)	2 (1.0)	2 (0.9)	
β-blockers				
Baseline	6 (18.75)	15 (7.8)	21 (9.4)	0.049‡
ES	6 (18.75)	10 (5.2)	16 (7.1)	0.006‡
Antihypertensive drugs				
Baseline	7 (21.9)	45 (23.4)	52 (23.2)	0.846‡
ES	5 (15.6)	49 (25.5)	54 (24.1)	0.226‡
Type 2 diabetes mellitus				
Baseline	5 (15.6)	32 (16.7)	37 (16.5)	0.883‡
Type 2 diabetes mellitus (medically treated)				
ES	1 (3.1)	17 (8.9)	18 (8.0)	0.270‡
Smoking	(*n* = 31)			
ES	6 (19.4)	55 (28.6)	61 (27.4)	0.282‡
Never smoked	15 (48.4)	72 (37.5)	87 (39.0)	0.249‡

Values in parentheses are percentages unless indicated otherwise; *values are mean (SD). *%TWL*, percentage total weight loss; *RYGB*, Roux-en-Y gastric bypass. †Independent samples *t* test, ‡*χ*2 test*. N*, number of patients. *ES*, end of study 10–15 years after RYGB

**Table 2:** SF-36 results at end of study

	Study group (≤ 50 bpm) (*n* = 32)	Reference group (> 50 bpm) (*n* = 192)	Total	*p*
SF-36 Domain *	(*n* = 31)	(*n* = 189–191)		
Physical function sum score	86.7 (16.3)	78.6 (21.5)	79.8 (21.0)	0.019†
Role physical sum score	75.8 (37.9)	60.5 (43.4)	62.7 (42.9)	0.048†
Bodily pain sum score	58.4 (25.6)	49.6 (28.9)	50.8 (28.6)	0.113†
General health sum score	72.5 (17.8)	66.1 (20.7)	67.0 (20.4)	0.105†
Vitality sum score	51.6 (22.6)	45.8 (21.4)	46.6 (21.6)	0.167†
Social function sum score	79.0 (22.7)	75.6 (26.8)	76.0 (26.3)	0.500†
Role EMOTIONAL sum score	83.9 (32.1)	77.3 (37.6)	78.2 (36.9)	0.355†
Mental health sum score	76.1 (14.6)	74.8 (16.1)	75.0 (15.9)	0.684†

Values in parentheses are percentages unless indicated otherwise; *values are mean (SD). *%TWL*, percentage total weight loss; *RYGB*, Roux-en-Y gastric bypass. †Independent samples *t* test, ‡*χ*2 test. *N*, number of patients; *ES*, end of study 10–15 years after RYGB

Supplementary data were retrospectively retrieved from hospital electronic health records, and were mainly baseline data: ECG, blood pressure, medication list (beta-blockers and antihypertensive drugs), and information regarding medical history. HR at baseline were retrieved by reviewing the patient’s pre-operative ECG.

### Statistical Analyses

Continuous variables are reported as means with standard deviation (SD), and independent samples *t* test was used to compare groups. Categorical variables are reported with numbers and percentages, and chi-squared tests were performed to compare groups. To check for normal distribution of continuous variables, QQ-plot was performed. Univariate and multiple linear regression analyses were used to investigate any relations between HR and selected parameters. Binary logistic regression was used to investigate the relationship between selected parameters and bradycardia (HR ≤ 50) at ES. All parameters with univariate *p* < 0.20 was tested in a forward model building. Two-sided *p* value < 0.05 was considered significant. The statistical analysis was performed using IBM SPSS version 28 (SPSS Inc., Chicago, IL, USA).

## Results

The mean HR among all the 546 patients in the BAROPS population was 66.3 (11.3) bpm. There was no between gender difference in HR in the total BAROPS population.

Thirty-two (6%) patients of the 546 patients in the BAROPS study had heart rate ≤ 50 bpm at follow up 12 years after RYGB and constitute the study group (SG). The comparator group (RG) (from 1 of 3 study hospitals) consisted of 192 patients. The mean HR at ES was 47.0 (2.8) bpm in the study group, and 66.4 (10.2) bpm in the comparator group (*p* < 0.001).

Baseline and ES characteristics are reported in Tables 1 and 2. There were 79.1% women in the total BAROPS (*n* = 546) population, and 78.1% women in the comparator group (*n* = 192). There were a larger proportion of men in the study group (37.5%) than in the comparator group (21.9%), but this difference was not statistically significant. The mean age pre-operative was 39.7 (8.8) years, and 51.4 (9.0) years at ES. The mean follow-up time for both groups was 12 (1.7) years. Mean BMI was 44.1 (5.2) kg/m^2^ pre-operative and 34.1 (6.5) kg/m^2^ at ES (*p* < 0.001).

The study group had a significantly reduced HR from baseline (67.6 (14.9) bpm) to ES (47.0 (2.8) bpm) with a 20 bpm drop 12 years after RYGB (*p* < 0.001). The reduction of HR in the comparator group was 4.8 bpm (*p* < 0.001). Mean overall HR in both groups together at baseline was 70.7 and 63.6 at ES, with a significant reduction of 7 bpm (*p* =  < 0.001).

Compared to the comparator group, a higher proportion of patients in the study group (18.8% vs. 7.8% pre-operative (*p* = 0.05), and 18.8% vs. 5.2% at ES (*p* = 0.006)) used beta-blockers.

Multiple regression analyses showed that %TWL between baseline and ES was negatively related to HR (*p* < 0.001), and smoking was positively related to HR (*p* = 0.014). The only significant predictor of bradycardia (HR ≤ 50) was the use of beta-blockers at ES (*p* = 0.010).

Furthermore, multiple regression analysis showed that there were two significant predictors of change in HR from baseline to ES: change in BMI from baseline to ES (*p* < 0.001) and baseline hypertension (*p* = 0.006).

There was no significant difference in the prevalence of dizziness or how the patients in the two groups rated their general physical or mental health. There was, however, a significant difference in SF-36 scores between the two groups related to physical health. The study group had a significantly higher score of the physical function domain (*p* = 0.019) and role physical domain (*p* = 0.048). The numeric values were generally better in the study group for all eight SF-36 domains, but only statistically significant for the physical domains.

## Discussion

The main findings of the study were a 6% frequency of bradycardia 12 years after RYGB, that heart rate and changes in heart rate after RYGB was linked to the amount of weight loss, and that bradycardia 12 years after RYGB was associated with better self-reported physical health. The only predictor of bradycardia in our study was the use of beta blocker.

Despite a larger reduction in HR in the study group, the number of patients using beta-blockers was the same both at baseline and ES. There were no significant differences between the groups regarding weight loss and blood pressure, or HR at the time of surgery. It may be that patients who have a larger change in HR are more sensitive to changes in weight as there was no change in the use of beta-blockers. We do not have information on the doses of beta-blockers at baseline and end of study. However, we do not have evidence to support the mechanism for this finding. To our knowledge, this is a novel finding as none of these relations have previously been described in a study with a mean follow-up of more than 10 years or with bradycardia defined as HR ≤ 50 bpm.

Only a few case reports [17, 21, 22], and one prior study [9] have been published to address this matter. Malik et al. were the first to demonstrate that bradycardia is a notable outcome of bariatric surgery, and its association with the reduction in BMI.

In contrast to our paper, Malik et al. demonstrated that 25 out of the 137 patients (18.2%) had postoperative bradycardia at rest ≤ 24 months after bariatric surgery, and that it resolved in 20 of 25 patients despite continued albeit decreasing weight loss. It should be noted that their study defined bradycardia as a HR < 60 bpm, with a mean postoperatively HR of 56.7 (3.9) bpm, whereas we defined bradycardia as a HR ≤ 50 bpm with a mean HR of 47 (2.8) bpm in the study group. Our study does not have data regarding HR at peak BMI reduction 1–2 years after RYGB. Based on Malik’s findings, we could assume that the number of patients with bradycardia in our study would have been higher at this time. However, this assumption requires confirmation by further research.

Previous research has shown that initial 10% weight loss in patients without obesity resulted in a decreased sympathetic tone and increased parasympathetic tone [23–25]. In our study, the mean weight loss in both groups from baseline to ES where 29.2 kg (22.9%), and we witnessed a reduced HR in these patients. The significant reduction in HR (7 bpm) 12 years after RYGB adds to the body of evidence of a significant relation between weight loss and heart rate. Whether this relationship is different for bariatric surgery and medical weight loss is unknown, and cannot be answered by the present study.

The mechanism behind the autonomic changes has been evaluated in a few studies [7, 25, 26]. Research indicates that the augmented parasympathetic tone may be due to the decrease in adipose tissue or regression of “fat mass disease” [27, 28], and further reduction in leptin levels [7, 10, 12, 29].

The prevalence of bradycardia defined as HR < 50 bpm in a healthy population has previously been described in two Norwegian studies conducted by Bjørnstad et al. in 1991 [30] and 1993 [31]. They investigated resting ECG in well-trained students matched with a control group of sedentary students and office workers with a mean age of 24.2 (4.3) years. There was a sex difference in HR of 4 bpm in both athletes and controls, and a between group difference of 6 bpm, with a mean HR of 68 bpm in the control group compared to 66 bpm in our study. Bjørnstad el al found a prevalence of bradycardia (HR < 50 bpm) of 7.3% in the control group and 17.8% in the athletes. Compared to our findings, the values in these two studies refer to younger and healthy subjects, but the prevalence in the control group is of the same magnitude as in our study.

Most studies regarding bradycardia after bariatric surgery conclude that symptomatic bradycardia is rare, and that it does not often require treatment [21]. Our findings regarding self-reported health-related quality of life between the groups support this. Our pre-study assumptions were that the reference group may score higher in self-reported health, and that the frequency of self-reported dizziness could be higher in the study group. However, there were slightly higher reports of dizziness in the reference group, and regarding self-reported health, the numeric values were overall higher in the study group, and significantly higher regarding self-reported physical health. We do not have supplementary data that could explain this somewhat unexpected phenomenon. However, this is an interesting observation, and it merits further exploration in future studies.

### Strengths and Limitations

The BAROPS population used in this study is a large, unselected population, and we have a long observation period of more than 10 years. The ES parameters were collected prospectively with virtually no missing data.

The observational design with only data from the ES control is, however, a limitation. To identify symptomatic bradycardia after bariatric surgery, a prospective longitudinal study to explore a cause-effect with data postoperatively at peak reduction in BMI, would have been of interest.

We have no population without bariatric surgery that is matched regarding age, gender, blood pressure, and smoking to compare the frequency of bradycardia. This would be of interest to define the effect of bariatric surgery itself, especially if also matched regarding BMI.

We have used a HR ≤ 50 bpm as definition of bradycardia in this study, and this differs from most other studies, and may be a limitation comparing findings across studies.

## Conclusion

This study contributes new knowledge regarding the long-term (12 years) relation between heart rate and weight loss after bariatric surgery. The frequency of bradycardia is low (6% HR ≤ 50) and is related to the use of beta-blockers. Long-term changes in heart rate after bariatric surgery is related to the amount of weight loss, and without any indication of increased symptom burden in patients with bradycardia. There is a need for further research regarding the mechanisms for this relation, and whether the relationship differs between different modes of weight loss.

## Data Availability

The data are not available due to restrictions related to the ethical approval for the study.
